# Porous Polymer Films with Tunable Pore Size and Morphology by Vapor Deposition

**DOI:** 10.1021/acsapm.2c01032

**Published:** 2022-09-20

**Authors:** Ni Huo, Sheng Ye, Andrew J. Ouderkirk, Wyatt E. Tenhaeff

**Affiliations:** † Department of Chemical Engineering, University of Rochester, Rochester, New York14627, United States; ‡ Facebook Reality Labs, 9845 Willows Rd, Redmond, Washington98052, United States

**Keywords:** porous polymers, thin film, coating, chemical vapor deposition, phase behavior

## Abstract

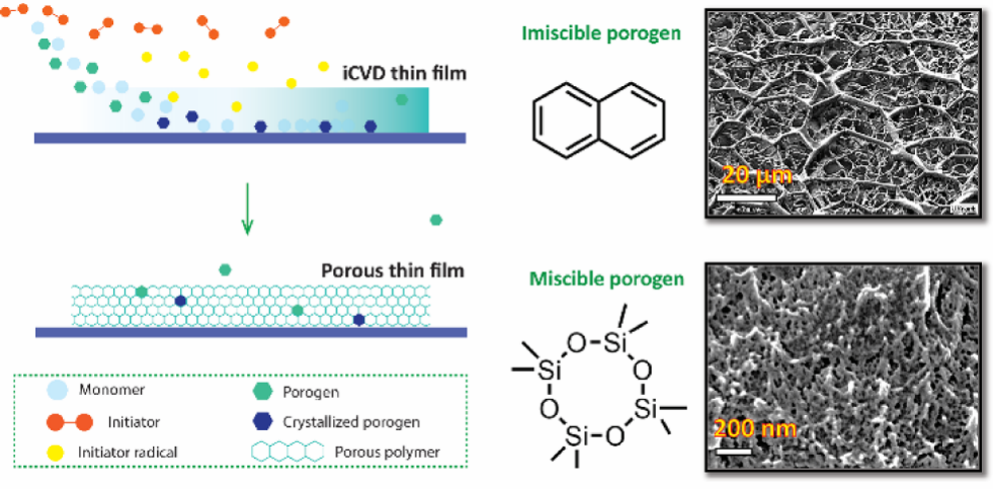

The fabrication of porous polymer thin films with precise thickness and morphological control through conventional solvent-based techniques is challenging. Herein, we present a fabrication method for porous polymer thin films based on chemical vapor deposition that provides control over pore size, pore morphology, and film thickness. The porous films are prepared by co-depositing crystallizable porogen molecules with cross-linked poly(glycidyl methacrylate) (pGMA) thin films, which are synthesized by initiated chemical vapor deposition (iCVD). As the porogen is deposited, it crystallizes and phase-separates from the polymer film; simultaneous polymerization of pGMA limits crystal growth, controlling the size of crystals. Using naphthalene as porogen resulted in thin films with pore sizes from 5.9 to 24.2 μm and porosities ranging from 59.4 to 78.4%. Using octamethylcyclotetrasiloxane as porogen, which is miscible with the GMA monomer, drastically reduced the pore dimensions, ranging from 14.4 to 65.3 nm with porosities from 8.0 to 33.2%. The film morphology was highly dependent on the relative kinetics of porogen crystallization, phase separation, and heterogeneous polymerization. The kinetics of these competing processes are discussed qualitatively based on nucleation theory and Cahn–Hilliard theory. Fourier-transform infrared spectroscopy confirmed the retention of the reactive epoxide functionality of glycidyl methacrylate, which can enable further chemical derivatization as required for application in optoelectronics, sensing, separations, and biomedical devices.

## Introduction

Porous polymeric thin films find wide application in the fields of microelectromechanical systems, optical interference coatings, tissue engineering, and biosensing. For example, they can be used as ultralow refractive index optical coatings,^1−3^ low *k* dielectric layers in microelectronics,^4,5^ diffusion layers for gas sensors,^6,7^ and electroactive polymers (EAP) incorporated into wearable haptic devices and virtual reality (VR)/augmented reality (AR) systems.^8,9^ Common fabrication techniques used to produce porous polymeric films include top-down approaches, such as nanoimprinting and track etching, as well as bottom-up approaches, such as block-copolymer self-assembly, electrospinning, and various phase inversion approaches, including polymerization-induced phase separation and thermally induced phase separation.^10−12^ While these well-developed methods provide excellent control over pore size, porosity, and chemical functionality, one limitation is the reliance on solvent. Solubility requirements limit the generalizability and controllability of these methods. The preparation of uniform, conformal porous coatings on complex, micro- and nano-structured nonplanar substrates is challenging due to surface tension effects of the solvent.

Recently, vapor-processing techniques—chemical vapor deposition, molecular layer deposition, and atomic layer deposition, for example—have been the subject of intense research interest and have found applications outside traditional semiconductor fabrication facilities due to their solventless nature and ability to conformally coat complex substrate geometries. Initiated chemical vapor deposition (iCVD) is a versatile and delicate technique used extensively to prepare polymeric thin films and coatings. It is a one-step polymerization method that readily coats both flat and irregularly shaped substrates with exceptional control over film composition—often identical to those obtained via conventional free radical polymerizations in solution.^13,14^

**Figure 1:**
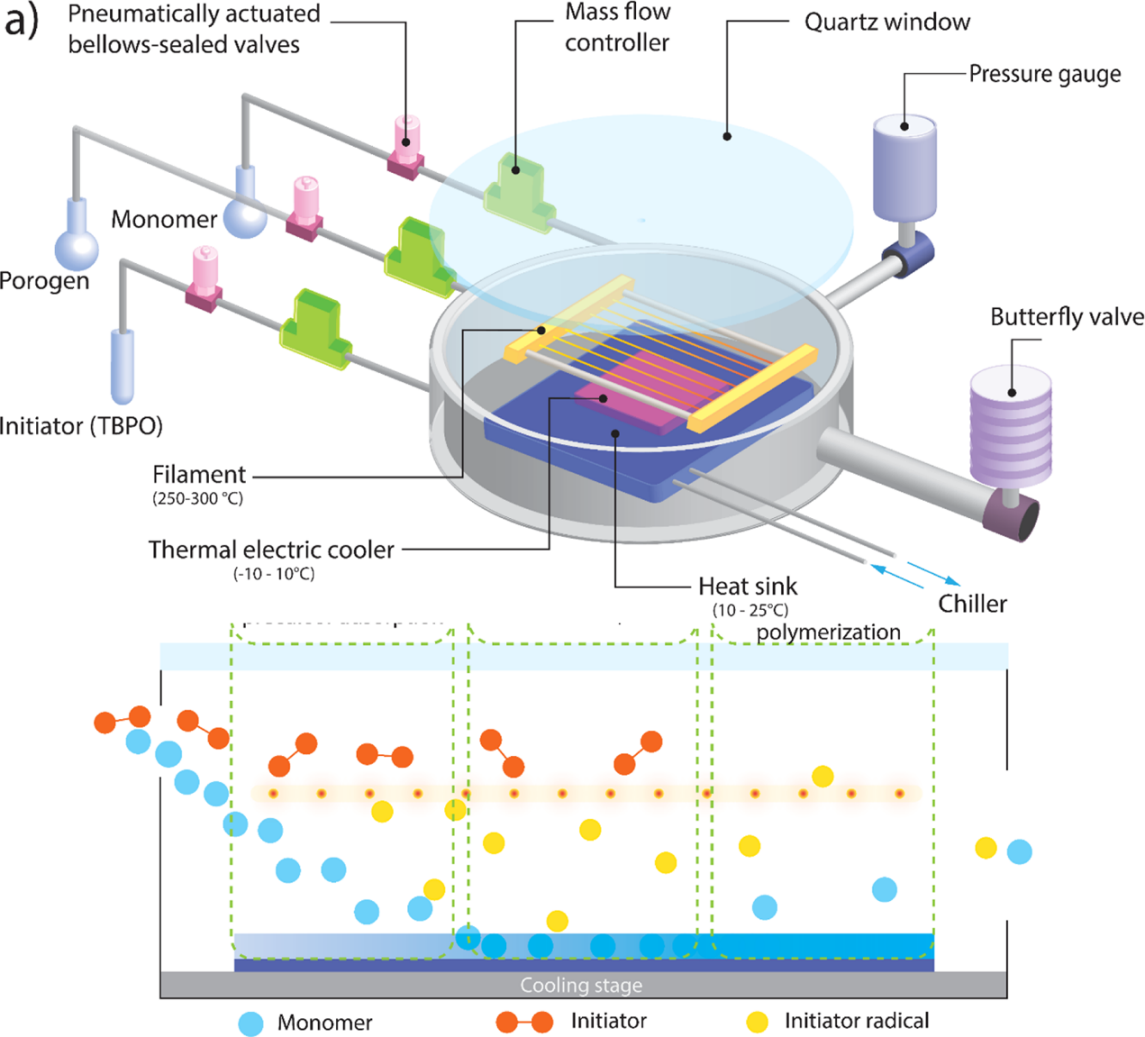
Schematic of (a) iCVD reactor and (b) depiction of the heterogeneous polymerization mechanism of iCVD.

Schematics of a customized iCVD reactor and the reaction process in the reactor are presented in Figure 1. Initiator and monomer vapors are delivered into a vacuum chamber maintained at a pressure between 100 and 1000 mTorr. Inside the chamber, a cooled stage promotes monomer adsorption to the substrate. Meanwhile, initiator vapor is thermally activated as it passes over a heated filament wire resistively heated to 200–350 °C and undergoes thermal homolysis to form radicals. The filament temperature is sufficient to cleave the labile peroxide bonds of the initiator while leaving the monomer species intact. These radicals collide with the surface-adsorbed monomers on the cooled substrate, triggering polymerization.^13^ The substrate temperature is adjustable and can be lower than room temperature. The low temperature of the substrate is crucial as it enables the coating of thermally sensitive materials, including polymers that would otherwise decompose or undergo thermal phase transitions at elevated temperatures—setting iCVD apart from many other vapor deposition techniques.^15,16^

Given these unique processing capabilities, iCVD is a compelling process for fabricating porous thin films employed as EAP layers in AR/VR display devices.^17^ Herein, the porous thin films are sandwiched between compliant electrodes supported on nonplanar, polymeric substrates; precise control over layer thickness and pore morphology is critical in this application.^8,9^ Several studies have emerged exploring the preparation of porous polymer films by iCVD. Dianat et al. prepared robust porous polymer membranes by first crystallizing then polymerizing the adsorbed monomer.^18,19^ The technique uses a single component—methacrylic acid in the initial studies—to function as both the sacrificial template and the polymerizable precursor. The process promotes adsorption and crystallization of a layer of methacrylic acid on the substrate, which then templates further film deposition. The deposited layers have pillar-like textured morphologies, dual-scale porosity, broad pore distributions, and thicknesses on the order of hundreds of micrometers. This process requires utilizing a monomer that possesses both a suitable freezing point for the templating process and desired chemical functionality to be incorporated into the final film composition. Using a different strategy, Tao and Anthamatten reported an open-cell macroporous membrane formed by phase inversion of a two-component system consisting of monomer and immiscible porogen, which further increases the degrees of freedom in precursor formulation.^20^ Due to the immiscibility between porogen and monomer, phase separation occurs immediately once the deposition commences and is gradually halted by cross-linking, generating a stiff polymeric network that arrests further phase separation. The porogen is then removed by thermal annealing in vacuo. The porous morphology appears as a network made of fused polymer spheres, with pore sizes ranging from hundreds of nanometers to several microns. The deposited layers also have thicknesses on the order of hundreds of micrometers.

However, in many applications of porous polymer coatings, such as microelectronic/semiconductor and optical devices, the desired film thickness is sub-micrometer. In optical interference coating designs, for example, the typical thickness of a single-layer anti-reflective(AR) coating is a quarter-wave optical thickness at the target wavelength.^21^ Depending on the refractive indexes of the coating and substrate, the appropriate thickness for minimizing reflectance can vary from tens to hundreds of nanometers.^21,22^ In addition, when porous polymeric coating is used as a low-refractive index layer, the pore size of these layers should be considerably smaller than the wavelength of light to prevent Mie scattering.^23^ Addressing these requirements, this study explores the potential of iCVD combined with phase separation to prepare porous polymer thin films with reduced thicknesses and nanoscale porosity. Crystallizable porogens of varying miscibility with the monomer were incorporated into the iCVD process. During the iCVD process, the vapor of the crystallizable porogen is introduced concurrently and deposits on the substrate simultaneous to polymer film growth. Through consideration of porogen miscibility, pore sizes ranging from several nanometers to tens of micrometers were prepared, distinguishing this work from previous studies. Specifically, macroporous polymeric coatings with well-defined geometric void shapes and dual-scale porosity are prepared using crystallizable naphthalene that is immiscible with the monomer, glycidyl methacrylate (GMA). Nanovoided coatings with diameters as small as 14.4–65.3 nm were prepared using miscible octamethylcyclotetrasiloxane (D4) porogen. Moreover, the film thickness of these nanovoided films is highly controllable; thicknesses from 1 to 25μm were achieved—closer to the required thickness for many applications. Furthermore, we demonstrated the preparation of porous film without cross-linking, further extending the generality of this approach.

## Results and Discussion

The experiments for the preparation of porous film using two distinct crystallizable porogens are discussed in this section. The two crystallizable porogens were selected based on their miscibility with GMA. The first was naphthalene, a porogen that is immiscible with GMA. The other was D4, a porogen highly miscible with GMA. GMA was selected because it is a common monomer used extensively in preparing porous polymers,^11^ and the polymerization kinetics of poly(glycidyl methacrylate) (pGMA) in the iCVD process is well established.^24,25^

### Depositions of Porous Thin Films Using Naphthalene as Porogen

Naphthalene is a white crystalline solid at room temperature, having a melting point of 80.2 °C. Initial experiments showed that naphthalene is immiscible with GMA monomer and pGMA polymer. Importantly, naphthalene has a vapor pressure (64.8 mTorr at 20 °C)^26,27^ comparable to GMA’s (600 ± 300 mTorr at 25 °C),^26^ which is convenient for controlling the relative surface concentrations of GMA and naphthalene. Naphthalene crystals readily sublime and thus can be eliminated from the deposited film without a solvent wash, introducing surface tension effects that cause void collapse. By co-depositing naphthalene and monomers, macroporous thin films with well-defined network structures were demonstrated.

Guided by previous studies, the primary variables explored in controlling the porosity in the polymer films were the relative surface concentrations of the precursors and polymerization temperature.^19,20^ In iCVD, the precursor concentration adsorbed to the substrate surface is positively correlated to its reduced partial pressure—the ratio of its partial pressure to its saturated vapor pressure evaluated at the substrate temperature, as shown in the formula below.
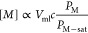
1where [*M*] is the concentration of the adsorbed monomer, *P*_M_ is the partial pressure of monomer vapor, *P*_M-sat_ is the saturated vapor pressure of the monomer defined by the substrate temperature, *V*_ml_ is the adsorbed volume of a monolayer of monomer, and c is a constant related to the enthalpies of desorption vaporization. Therefore, *P*_M_/*P*_M-sat_ and *P*_P_/*P*_P-sat_ were a proxy for surface concentrations of monomer and porogen, respectively, and used as convenient control parameters.

Initially, a series of experiments were conducted to screen the wide parameter space available to iCVD to identify a practical process window over which porous thin films can form. Generally, it was critical that the rate of naphthalene deposition and phase separation was comparable to the rate of film polymerization. If the rate of naphthalene crystallization was large relative to polymerization, the substrate became covered with macroscopic naphthalene crystals, and a continuous polymeric film did not form over the entire substrate. On the other hand, if insufficient naphthalene was introduced, a dense film of pGMA was deposited with negligible naphthalene inclusion. Three critical processing conditions for reliable formation of porous films over the entire Si substrate were found based on these experiments.^20^ First, controllable void formation occurs when the ratio of reduced partial pressures (*P*_P_/*P*_P-sat_)/(*P*_M_/*P*_M-sat_) was roughly 4:1. Second, naphthalene must be oversaturated (*P*_P_/*P*_P-sat_ > 1) to ensure naphthalene incorporation in the film. Third, due to the large flows of naphthalene and rapid deposition, the monomer must also be oversaturated to increase its surface concentration for rapid film growth kinetics. Initially, a substrate temperature (*T*_sub_) of 5 °C was adopted to satisfy the three conditions above. However, massive naphthalene crystallization was observed for depositions conducted below 5 °C, preventing the formation of a continuous polymer film (see Figure S1a). Giant crystals formed because the naphthalene’s crystallization was considerably faster than GMA polymerization at low *T*_sub_.

To avoid the vast crystal growth, a higher *T*_sub_ (10–20 °C) was adopted, along with increased pressure to maintain *P*_P_/*P*_P-sat_ and *P*_M_/*P*_M-sat_ at elevated *T*_sub_. The experiments showed that when *T*_sub_ was increased to above 10 °C, massive crystallization of naphthalene was effectively restrained, and porosity was generated in the film. However, the pores were relatively ill-defined and did not have faceted boundaries as would be expected of crystallites (see Figure S1b). Because GMA and naphthalene were both supersaturated during the deposition process, they gradually formed a liquid layer on the chilled substrate (observed visually during the process). This observation suggested that naphthalene did not crystallize even though 10 °C was far below its melting point. The suppressed crystal growth can be explained by the impurity effect.^28^ The critical supersaturation condition for naphthalene crystals to form is affected by the concentration of other precursors and polymers formed.^29^ Given that naphthalene stayed in the film as a liquid, the morphology of voids was not fixed during the curing process, leading to ill-defined voids. Although void formation was demonstrated, porous films with defined morphologies were not achieved.

**Figure 2:**
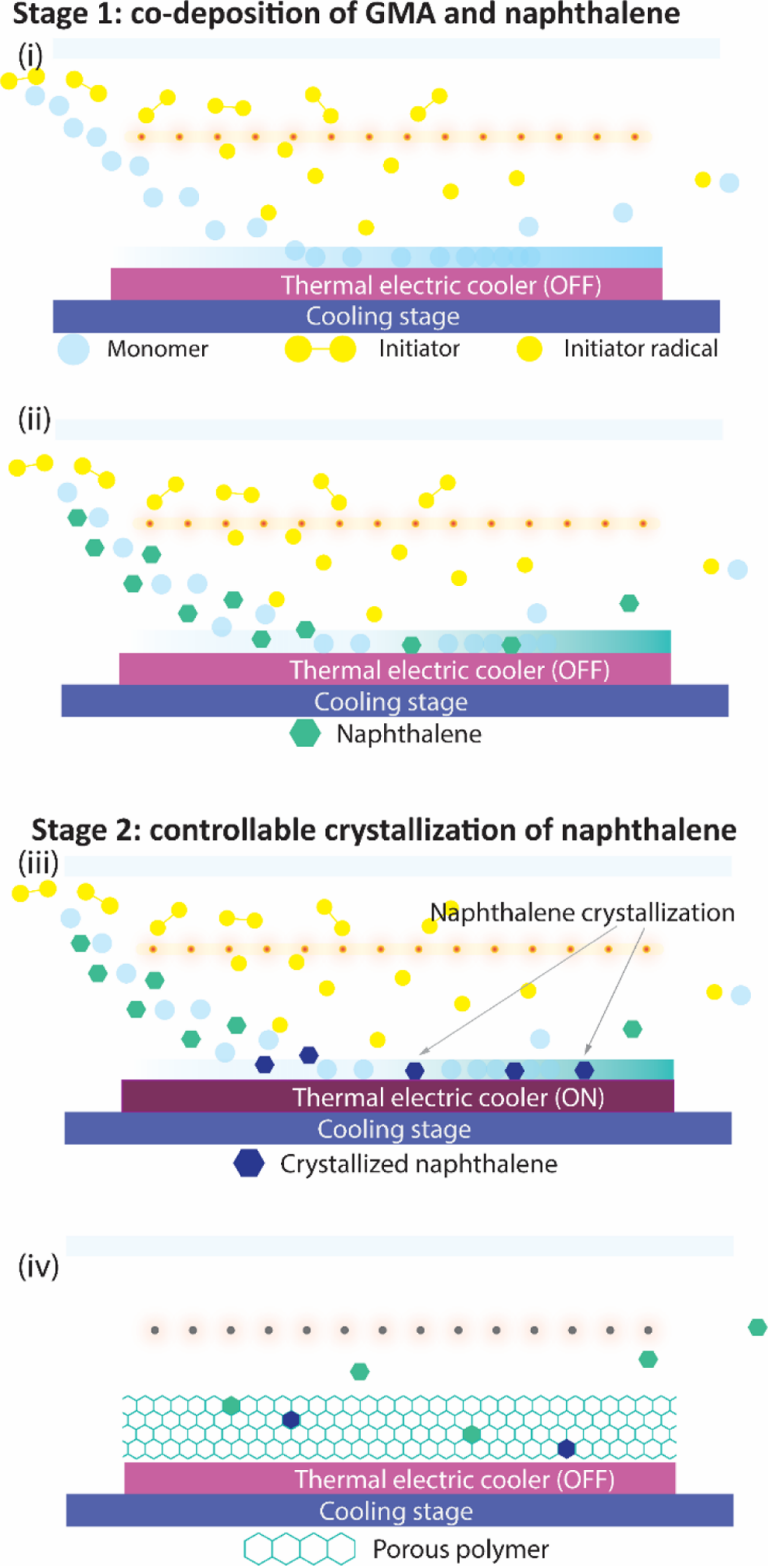
Schematic illustrations of two-stage porous film deposition process with iCVD and thermal shock, consisting of (i) deposition of pGMA, (ii) co-deposition of pGMA and naphthalene, (iii) crystallization of naphthalene under thermal shock, and (iv) sublimation of naphthalene under vacuum.

For control over crystal size and size distribution, the polymerization rate of GMA and the crystallization rate of naphthalene must be balanced. To achieve this, the deposition was divided into two stages, as conceptualized in Figure 2. During the first stage, the substrate was maintained at 10 °C. A 100 nm pGMA base layer was deposited to prevent the dewetting of the following layer. Saturated vapors of naphthalene and GMA fed into the chamber formed a liquid mixture layer where naphthalene was uniformly seeded in advance of the massive nucleation of naphthalene. At this stage, the relatively higher *T*_sub_ (10 °C) inhibited the crystallization of naphthalene but not polymerization, which prevented the formation of giant crystals. During the second deposition stage, a thermal shock was introduced to stimulate the crystallization of naphthalene. Thermal shock in this work refers to a rapid temperature drop of the deposition substrate from 10 to 5 °C or lower. It provided additional supercooling that compensated for the free energy penalty of forming surfaces, thus promoting the nucleation and crystallization of naphthalene. The thermal shock process was achieved via embedding a thermal electric cooler in the chill plate, as illustrated in Figure 2. Under temperature shock, the liquid film composed of GMA and naphthalene was supercooled and entered a supersaturated state. Once the critical supersaturation condition for crystallization was reached, massive nucleation occurred almost instantaneously, followed by the formation of crystals with uniform sizes. As polymerization proceeded, these crystals were then segregated by the growing polymer chains, eventually leading to well-defined voids with more uniform sizes (see Figure S1c). Compared with previous results using high *T*_sub_, the samples prepared with thermal shock exhibited a tunable void size and a defined void shape. Therefore, thermal shock was demonstrated to be an effective way of triggering simultaneous nucleation and was adopted to control void size and morphology.

After exploring primary conditions for pore formation and refining the processing approach, a systematic set of experiments was conducted to study the effect of processing conditions on film morphology. Our results indicate that thin films with 3D web-like structures and pore diameters ranging from 5.9 to 24.2 μm can be formed by altering the thermal shock temperatures (*T*_TEC_). Four conditions were selected for demonstration purposes, and the critical experimental conditions are listed in Table 1. The only independent control variable of these four samples was *T*_TEC_. Sample SP-N1 was deposited without thermal shock and served as a control sample. For consistency, the deposition time was limited to 10 min for all samples. The volumetric flow rates of ethylene glycol diacrylate (EGDA), GMA, and naphthalene were maintained at 0.4, 2.5, and 5.0 sccm, respectively. In the first deposition stage, the substrate temperature remained at 10 °C for all samples. The second deposition stage was thermal shock, where the substrate temperature varied from −5 to 5 °C.

**Table 1:** Key Deposition Parameters for the Fabrication of Porous Polymer Films Using Naphthalene as Porogen^a^

	*P*/*P*_sat_					
sample ID	EGDA	GMA	Naph	Naph/GMA	*T*_sub_ (°C)	*T*_TEC_ (°C)
SP-N1	1.21	1.1	4.5	4.1	10	10
SP-N2	1.21	1.1	4.5	4.1	10	5
SP-N3	1.28	1.47	6	4.1	10	0
SP-N4	1.31	1.75	7	4	10	–5

a Naphthalene and GMA’s surface concentration are dependent variables that change with thermal shock temperature.

**Figure 3:**
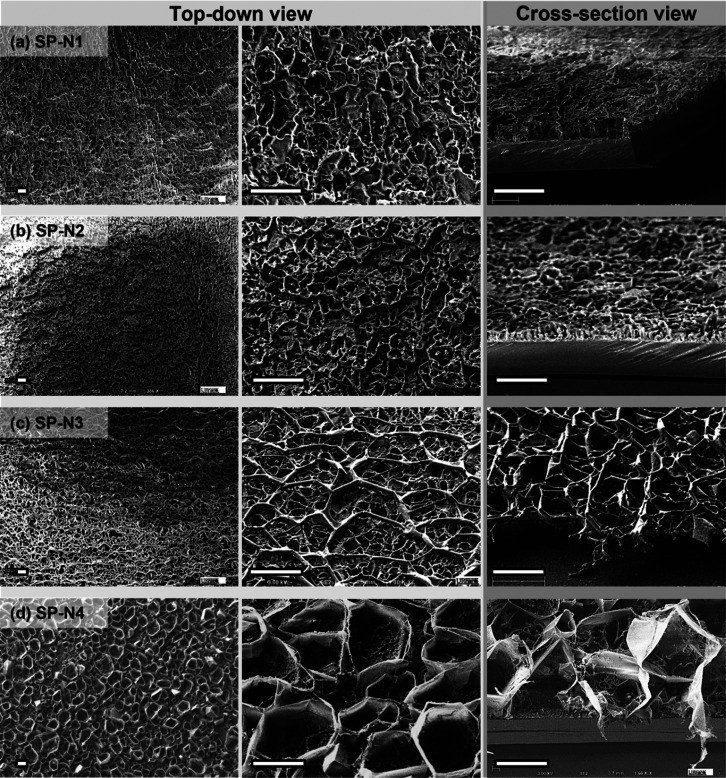
Top-down and cross-sectional SEM images of vapor-deposited polymer using naphthalene as a porogen. The thermal shock temperatures were (a) 10, (b) 5, (c) 0, and (d) −5 °C and correspond to entries in Table 2. All scale bars represent 20 μm.

Top-down and cross-sectional scanning electron microscopy (SEM) images of the films provided in Figure 3 reveal the effect of the thermal shock on pore morphology. SP-N1 was prepared at a fixed substrate temperature and possessed an ill-defined, highly size-disperse pore morphology. SP-N2, which introduced a 5 °C thermal shock, had more defined pore outlines (see Figure 3b), suggesting that these pores were templated by crystallized naphthalene. Because the thermal shock was the only difference between SP-N1 and SP-N2, it is believed that thermal shock stimulated the nucleation of naphthalene crystallites. A qualitative comparison of the SEM images in Figure 3 reveals that, as the shock temperature is reduced, the void dimensions grow and the void outlines become more defined. This observation is consistent with well-established findings that increased supersaturation will promote nucleation and crystallization.^30^ SP-N4, prepared with the lowest thermal shock temperature of −5 °C, had the largest average void dimension. The crystal size was determined predominantly by crystallization and polymerization kinetics of naphthalene and GMA, respectively. Crystal growth was faster at lower thermal shock temperature due to the increased supercooling and degree of supersaturation (*P*_P_/*P*_P-sat_). Meanwhile, the polymerization kinetics of GMA were expected to decrease at these low temperatures. Thus, it is possible that the polymer did not fully cure and develop its full mechanical properties during this phase separation process, allowing the crystals to grow at the expense of the polymer volume.

**Figure 4:**
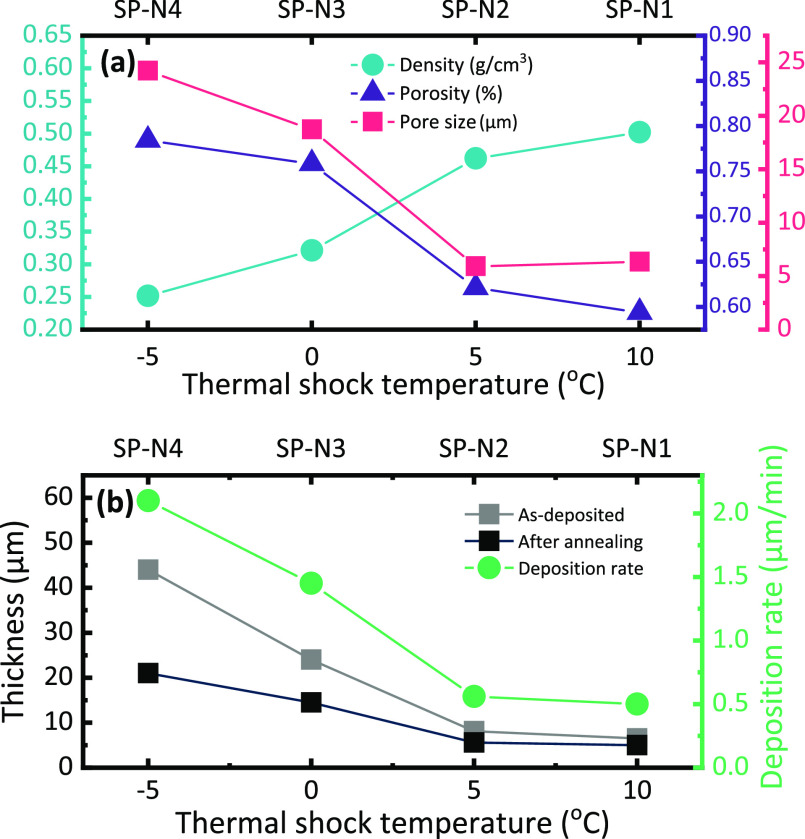
(a) Density, porosity, and pore size of samples deposited using naphthalene as porogen as a function of thermal shock temperature. (b) Sample thickness and deposition rate as a function of thermal shock temperature.

For quantitative analysis of the film morphologies, the porosity of the films was calculated by implementing image analysis of the SEM micrographs using image-processing algorithms.^31^ The processed SEM images for quantifying pore size and porosity as well as the pore size distribution histograms are presented in Figures S2 and S3. As indicated in Figure 4a, SP-N4 exhibits the highest porosity of 78.4%, whereas SP-N1 exhibits the lowest porosity of 59.4%. The sample porosities showed an obvious increasing trend with decreasing temperature. In addition, the measured density of the porous films varied from 0.25 to 0.50 g/cm^3^, which was significantly lower than that of dense, non-voided pGMA prepared by iCVD (1.07 g cm^3^).^27^ Given the limited sample volume and micrometer-scale pore diameters, SEM was best suited for analysis of film morphology. While other techniques, such as BET, glancing small angle X-ray scattering, and ellipsometric porosimetry, can provide pore information from the entire film volume, their applications to these films were infeasible for various reasons.^41^ It is important to recognize the possibility that the reported pore sizes and porosity are biased by the surface morphologies observable by SEM, but given the size of the pores relative to the film thickness, they are likely representative of the entire film.

Furthermore, the measured densities also showed a positive correlation with temperature, which is consistent with the trend in porosity. These trends in both porosity and density can be explained in terms of the combined effect of naphthalene crystallization rate and polymerization rate. Under lower *T*_TEC_, the increase in naphthalene’s crystallization rate resulted in larger crystallites occupying more film volume, which later became voids. In addition, the propagation rate of polymerization is dependent upon *T*_TEC_. Thus, the rate of polymerization decreases with lower *T*_TEC_, and consequently, less polymer films are produced at the same time as crystallites are coarsening and growing larger. Furthermore, due to the reduced polymerization kinetics, incomplete conversion of the adsorbed monomer to polymer is possible, and residual monomer is eliminated from the film in post-deposition annealing, which also contributed to the reduction in film density.

Observed trends in deposition rates and film thicknesses in Figure 4b provide further indirect evidence of naphthalene incorporation. The calculated deposition rates of all four samples were significantly larger than the maximum achievable deposition rates of pGMA (200 nm/min), suggesting that simultaneous naphthalene deposition was responsible for the increased deposition rate. In addition, the deposition rate was negatively correlated with *T*_TEC_, reaching a maximum of >2 μm/min at *T*_TEC_ = -5 °C, indicating that more naphthalene was incorporated at lower temperatures as the rate of naphthalene deposition and crystallization increased. Furthermore, sample thicknesses before and after post-deposition annealing are plotted in Figure 4b. In each case, the film thickness was reduced after thermal annealing, with the largest reduction of 52.3% observed for SP-N4, which had the lowest thermal shock temperature of −5 °C. Similar to the deposition rate, the thickness reduction is also negatively correlated with *T*_TEC_. It is concluded that the thickness reduction results from naphthalene sublimation during the annealing process, combined with some collapse of the highly porous film structure.

**Figure 5:**
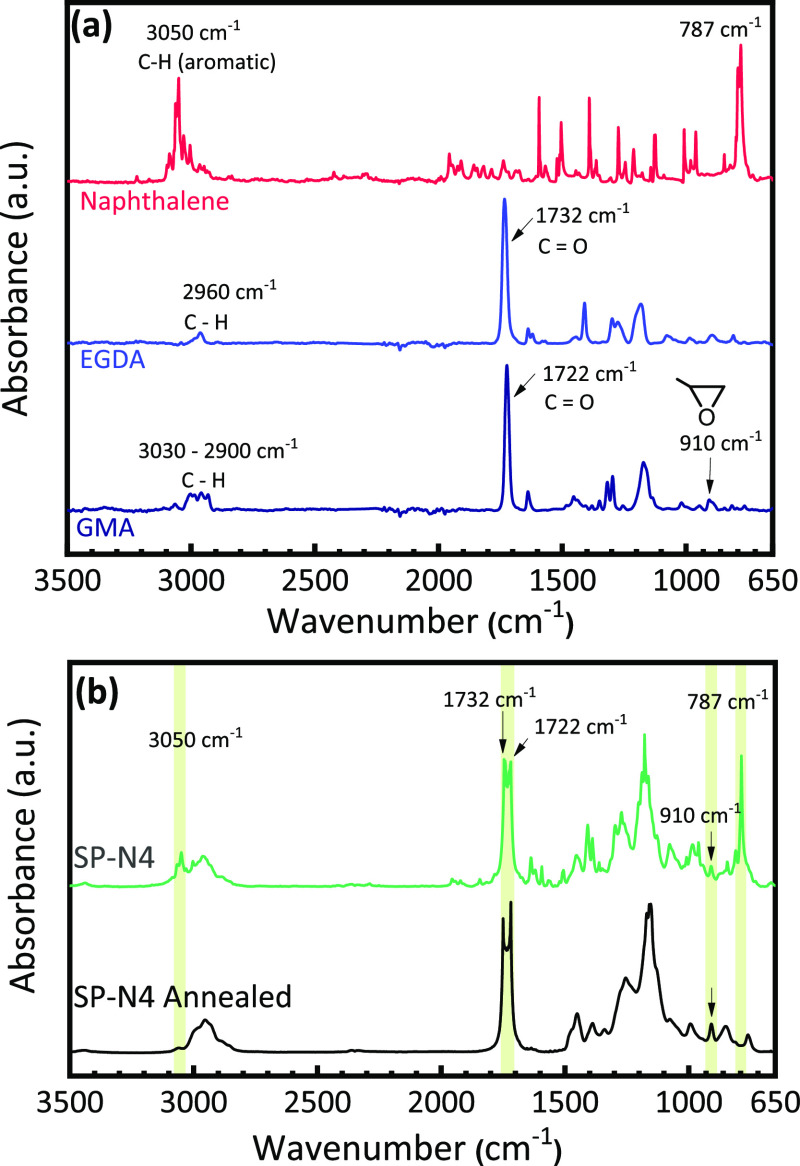
FTIR spectra of (a) GMA, EGDA, and naphthalene precursors and (b) as-deposited SP-N4 and annealed SP-N4 films. Characteristic vibrational modes of naphthalene and monomers are highlighted in the polymer film spectra.

Fourier-transform infrared (FTIR) spectroscopy was used to further detect the presence of naphthalene in the polymer film and confirm the polymerization of GMA. Figure 5a provides FTIR spectra of the precursors (GMA, EGDA, and naphthalene), and Figure 5b provides the spectra of sample SP-N4 pre- and post-thermal annealing. Two prominent, characteristic vibrational modes of naphthalene at 3050 and 787 cm^–1^ were identified and were well separated from other vibrational modes of the other precursors. The 3050 cm^–1^ mode is attributed to aromatic C–H stretching, while the 787 cm^–1^ band is assigned to C–C stretching modes and C–C–C out-of-plane vibrations, which are characteristic of an ortho-substituted benzene structure.^32^ These characteristic modes of naphthalene are apparent in the spectra of as-deposited SP-N4 (prior to thermal annealing to remove porogen) but are absent in the annealed film. This indicates that naphthalene is incorporated into the film during the iCVD process but can be fully removed through sublimation via a modest thermal annealing process. The FTIR spectra of the polymers also provide insights into the chemical structure of the deposited films. Comparing the spectra of SP-N4, pure GMA, and EGDA, the absorption peaks centered at 1722 and 1732 cm^–1^ are observed in SP-N4, which correspond to the carbonyl stretching vibration of the ester moieties in EGDA and GMA, respectively.^33^ This indicates that polymer films were successfully cross-linked through this iCVD process. Judging by the comparable intensities of the carbonyl peaks representing EGDA and GMA, SP-N4 is highly cross-linked. Based on reported *P*/*P*_sat_ in Table 1, SP-N4 has the lowest cross-linking density as it has the lowest ratio of EGDA to GMA surface concentrations during deposition. Therefore, all four samples are highly cross-linked. It is believed that the slight difference in cross-linking degree should not significantly impact the morphology of the porous film.

Importantly, polymerization can be verified through the disappearance of the C=C stretch modes of the vinyl monomer at *ca.* 1630 cm^–1^ in the polymer spectra.^33^ The peaks centered at 751 and 1161 cm^–1^ were attributed to the stretching vibrations of C–O–C and C–O in EGDA and GMA, respectively,^33^ suggesting that monomer structures are intact. The absorption bands centered at 908 cm^–1^ in the film spectra are associated with the epoxy moieties of GMA,^33^ revealing that the reactive functionality of GMA is retained during this process. The highly epoxide ring can be used as a convenient target for further derivatization as required in numerous applications.^19,33,34^

### Depositions of Porous Films Using D4 as Porogen

Macroporous polymeric thin films with voids on the order of several micrometers were formed using naphthalene. Experiments revealed that the competition between phase separation and polymerization is a key factor controlling pore size and porosity. Reducing the kinetics of crystallization and phase separation should lead to smaller void dimensions, which, according to Cahn–Hilliard theory, can be achieved by increasing the miscibility of the porogen within the monomer.^35^ Therefore, using a highly miscible yet crystallizable porogen like D4 should lead to a smaller void size.

A series of initial trials were conducted to explore the wide parameter space to form voided films with D4. These trials revealed a similar finding to the naphthalene case. Voids develop in the polymer film only under certain ratios of reduced partial pressures (precursor surface concentrations). With D4, the ratio was (*P*_P_/*P*_P-sat_)/(*P*_M_/*P*_M-sat_) = 1.7. Accordingly, subsequent experiments were conducted by fixing this ratio and examining the effect of substrate temperature. Due to the high vapor pressure of D4 (933 ± 46 mTorr at 25 °C),^36^ it was difficult to reach *P*/*P*_sat_ > 1 under practical deposition conditions for iCVD; thus, all depositions were conducted with D4 and GMA below saturation conditions (*P*/*P*_sat_ <1).

Distinct from the deposition process using naphthalene as porogen, thermal shock was not required to create voided films with D4. The kinetics of crystal growth with D4 was slower, and macroscopic crystallization was not observed as was the case with naphthalene. Thus, the effect of a single substrate temperature used throughout the deposition process on void morphologies was explored. Table 2 provides the key experimental conditions for the depositions with a fixed partial pressure ratio and using the substrate temperature as the independent variable. SP-D4 was prepared based on the same conditions as SP-D3, but EGDA cross-linker was included in the precursor mixture. For consistency, the deposition time was limited to 5 min for all samples, and the volumetric flow rates of GMA and D4 were maintained at 2.5 and 12 sccm, respectively.

**Table 2:** Key Deposition Parameters for the Fabrication of Porous Polymer Films Using D4 as Porogen

	*P*/*P*_sat_				
sample ID	EGDA	GMA	D4	D4/GMA	*T*_sub_ (°C)
SP-D1	0	0.13	0.2	1.8	18
SP-D2	0	0.16	0.3	1.8	12.8
SP-D3	0	0.27	0.5	1.7	8.6
SP-D4	0.1	0.27	0.4	1.7	8.6

**Figure 6:**
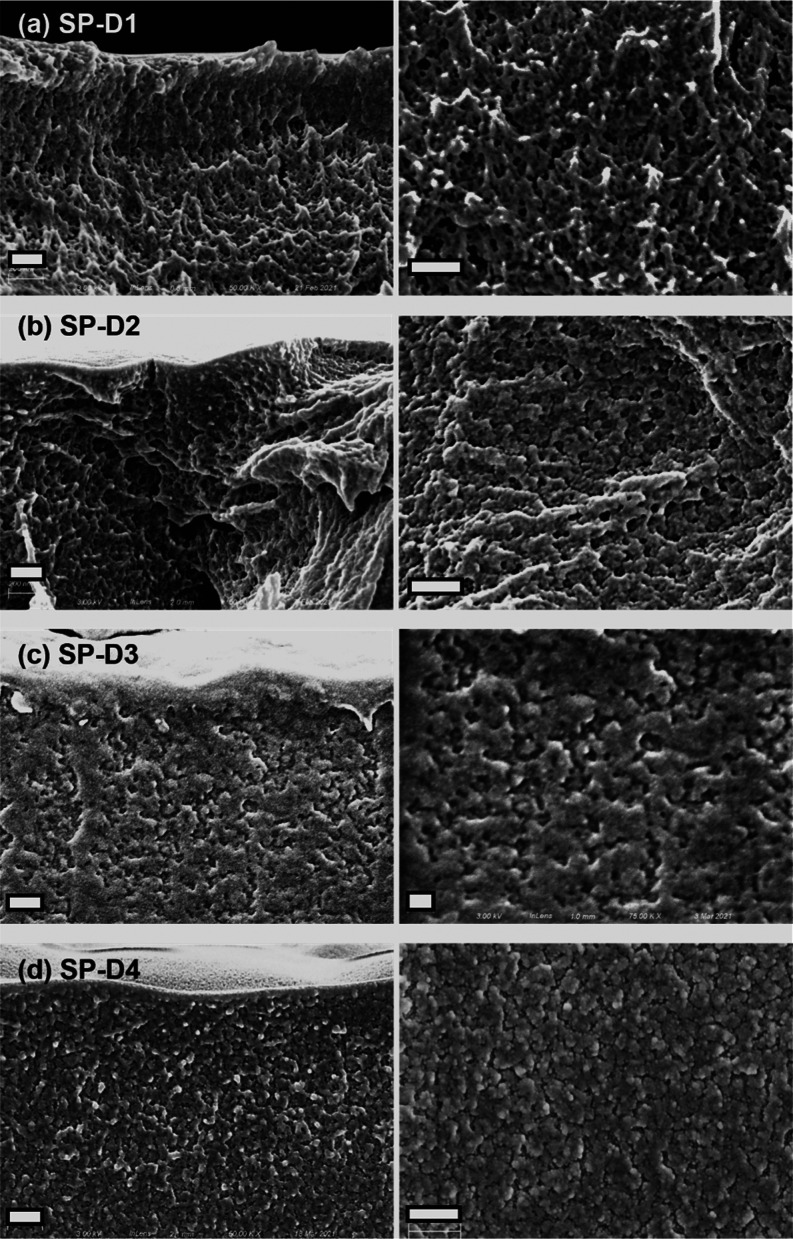
Top-down and cross-sectional SEM images of vapor-deposited polymer grown using D4 as porogen. Each sample was deposited at (a) 18.0, (b) 12.8, (c) 8.6, and (d) 8.6 °C (with cross-linker). All scale bars represent 200 nm.

Figure 6 provides the SEM images of the film morphology for all four samples, immediately revealing the effect of porogen miscibility on pore dimensions. Compared with the naphthalene case, the pore sizes generated using D4 were 1 to 2 orders of magnitude smaller due to the miscibility differences of D4 and naphthalene in GMA. In contrast to naphthalene, the phase separation of D4 and GMA in a certain temperature range remains slow, which eventually resulted in pores as small as tens of nanometers. This is believed to be among the smallest pores demonstrated in iCVD polymer thin films. Another paper by Trujillo et al. reported the preparation of porous polymer film using iCVD.^40^ Although no pore size information is provided, the ellipsometric analysis suggests little incoherent light scattering off the sample and pore sizes below 100 nm (below the threshold for significant scattering).

Judging by the void morphologies, D4 did not crystallize during the deposition. Given that D4 and GMA are soluble with each other, this can be explained by the freezing point depression. The maximum temperature difference between *T*_sub_ and D4’s melting point is around 9.4 °C, which is too small to compensate for the decrease in melting point of D4 in the mixture due to the impurity effect.^28^ Potentially, D4’s crystallization could be triggered by a low enough thermal shock temperature. However, an important trade-off to consider is the further reduction in polymerization kinetics, which is already compromised by the existence of D4. Thus, the study of D4 mainly focuses on the effect of *T*_sub_ on void size and morphologies.

**Figure 7:**
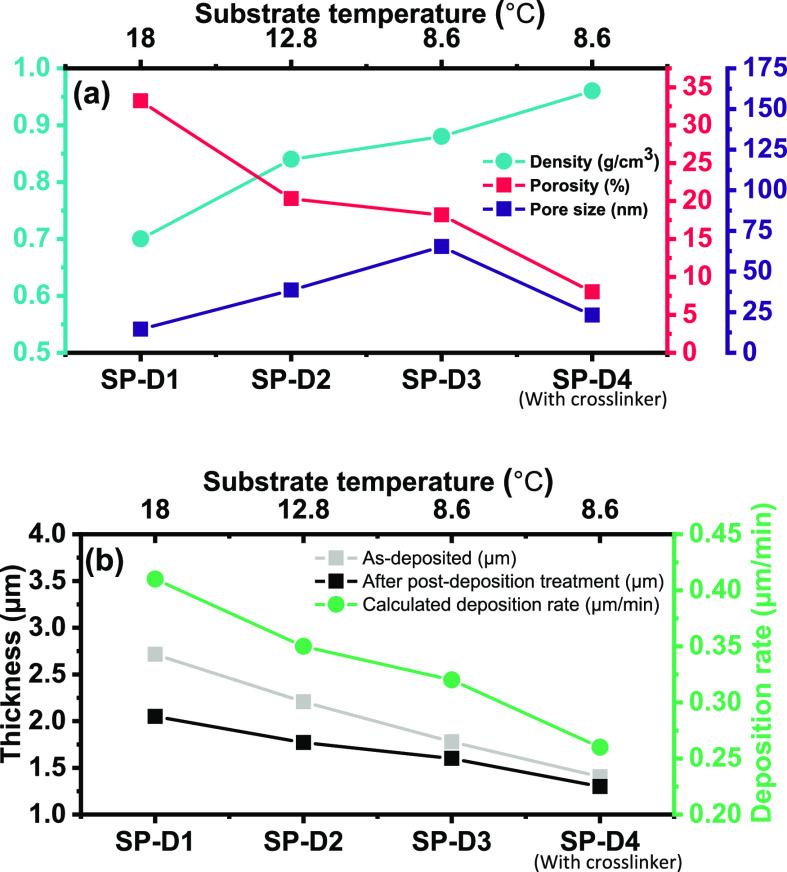
(a) Density, porosity, and pore size of samples deposited using D4 as porogen as a function of substrate temperature. SP-D4 is deposited with EGDA cross-linker. (b) Sample thickness and deposition rate as a function of substrate temperature.

The average pore sizes of SP-D1 to D4 are plotted in Figure 7a. All four samples prepared using D4 as porogen had average diameters of less than 100 nm, which were directly correlated to *T*_sub_. For SP-D1 (18.0 °C), the void diameter is as small as 14.4 nm, while for SP-D3 (8.6 °C), the diameter increased to 65.3 nm (Figure 7b). Consistent with the naphthalene case, lowering *T*_sub_ resulted in larger average pore dimensions. In the naphthalene case, the negative correlation between *T*_TEC_ and pore size can be explained by the combined effect of faster crystallization and slower polymerization. However, in the D4 case, the similar observation can be understood by the increased surface concentrations of porogen and monomer. The exact trend in polymerization rate cannot be fully resolved in the presence of D4, given the competition between the reduced propagation rate constant and increased monomer surface concentration. As *T*_sub_ decreases, the mobility of monomers is greatly reduced, potentially resulting in slower polymerization. On the contrary, the surface concentrations of both D4 and GMA increase proportionally when lowering *T*_sub_, resulting in faster polymerization. Considering the trend observed in Figure 6, the surface concentration of D4 appears to be the dominant factor for the void size change. In addition, the importance of polymerization kinetics can be appreciated through a comparison between SP-D3 and SP-D4. A minute concentration of EGDA cross-linker was included in SP-D4, which increases the polymerization kinetics due to the diacrylate functionality, suppresses phase separation, and ultimately results in a much smaller pore size of 23.1 nm.

The densities, porosities, and pore sizes of SP-D1 to D4 are plotted in Figure 7a. The processed SEM images for quantifying pore size and porosity as well as the pore size distribution histograms are presented in Figures S4 and S5. Film densities were measured using the same method described for naphthalene. Figure 7a reveals that the film densities measured by microbalance and the porosities calculated through image analysis algorithm are in good agreement. Notably, the densities of all four samples were lower than that of a pure, dense pGMA film and were negatively correlated to *T*_sub_. Correspondingly, the porosity of all samples was positively correlated to *T*_sub_. Interestingly, in contrast to the change in pore size, porosity is observed to decrease with *T*_sub_. This can be interpreted by the collapse of voids during annealing process as well as the reduced phase separation rate. Given that there is no cross-linking in the first three samples, the polymeric framework composed of pure GMA could not withstand atmospheric pressure and partially collapsed, resulting in reduced porosity. Also, a portion of deposited D4 that did not undergo phase separation remained in the polymeric phase, which further brings down the modulus of the polymer framework. The porosity difference in SP-D3 and SP-D4 is due to the accelerated polymerization kinetics through the inclusion of difunctional EGDA.

Figure 7b shows the calculated deposition rates of all four samples and the thickness change before and after the thermal annealing. The deposition rates were all larger than the maximum deposition rate of pure pGMA under similar deposition conditions (200 nm/min), confirming that D4 was incorporated into the film and increased the film thickness. Contrary to the results of naphthalene, the deposition rate is inversely correlated with *T*_sub_, suggesting that more D4 is trapped in the growing polymer matrix when *T*_sub_ is higher. In addition, thickness reduction was also observed after annealing (see Figure 7b), attributed to the elimination of D4 porogen and the collapse of polymer matrix during the annealing process. Similar to the deposition rate, the thickness reduction was also positively correlated with *T*_TEC_. The thickness reduction was 25.4% for SP-D1 (18.0 °C) while only 7.6% for SP-D4 (8.6 °C), again indicating that a larger volume of D4 was incorporated into SP-D1. Both trends observed in deposition rates and thickness change matched the trend of porosities.

**Figure 8:**
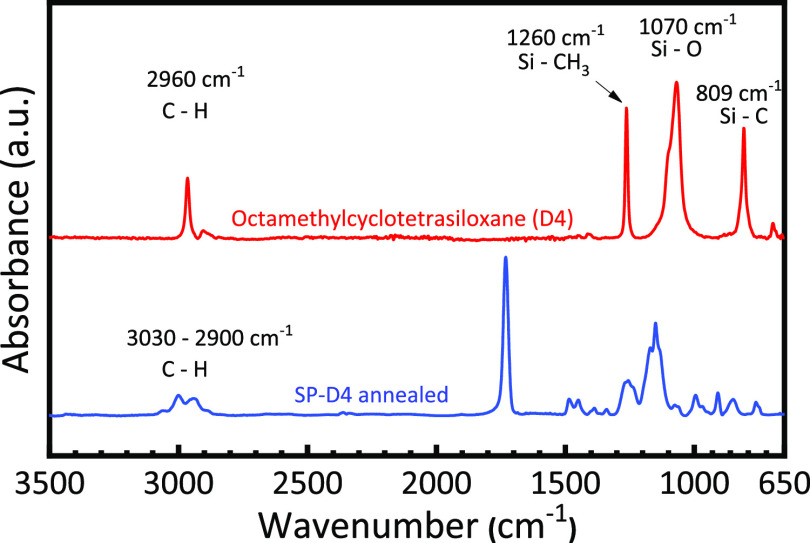
FTIR spectra of D4 porogen and annealed SP-D4 film.

FTIR spectroscopy was used to confirm the successful removal of D4 in the polymer film. The spectra of D4 and annealed sample SP-D4 are shown in Figure 8. D4 exhibits four main absorption peaks. The highest peak is located at 1070 cm^–1^, which can be attributed to Si–O stretching vibration in cyclic compounds (e.g., D4).^37−39^ The peak at 1260 cm^–1^ is assigned to the methyl rocking vibration from the Si–CH_3_ rock.^37−39^ The peak centered at 809 cm^–1^ is attributed to Si–C stretching.^37−39^ The peak centered at 2960 cm^–1^ corresponds to C–H methyl stretching.^37−39^ The absence of these characteristic peaks of D4 in the spectrum of annealed SP-D4 annealed post-deposition reveals that the residual D4 was successfully eliminated from the film. In fact, the majority of D4 was sublimed and removed from the film during sample transfer. D4 is relatively volatile and has rather high vapor pressure and low surface tension (18.4 ± 5.0 dyne/cm), making it easy to sublime. In the FTIR spectrum of SP-D4 after deposition (see Figure S6), there is no clear sign of D4 in the film. Because the iCVD chamber is evacuated after deposition and before sample removal, it is possible that D4 is sublimed and removed from the film under the reduced pressure.

## Conclusions

A straightforward vacuum deposition technique for fabricating porous polymer thin films has been demonstrated. The method relies on the co-deposition and phase separation of crystallizable porogens simultaneous to polymer thin film growth by iCVD. The pore size and morphology, in addition to the overall porosity, are primarily controlled by the substrate temperature and surface concentrations of the porogen and (co-)monomers. Using naphthalene and D4 porogens, which possess differing miscibilities with GMA, it was shown that the miscibility and melting point are critical design parameters that control pore morphologies. In the case of naphthalene, a two-step deposition process employing thermal shock is also critical to suppress macroscopic crystallization and seed the film with smaller domains, which crystallize and phase-separate with smaller dimensions (tens of micrometers). Using D4, pores with characteristic length scales on the order of tens of nanometers can be prepared, which are among the smallest pores achieved by iCVD.^40^ These results guide future development of porous polymer thin films where the porogen with different physical properties can be carefully selected to tune pore morphologies. It is also expected that this approach is amenable to a wide array of vinyl monomers possessing distinct pendant chemical functionalities to enable applications in biomedicine, separations, optoelectronics, and many other fields.

## Experimental Section

The following chemicals and materials were purchased and used as received: D4 (Gelest, 99% purity), naphthalene (Home Depot, 99.95% purity), GMA (Sigma-Aldrich, 97% purity), EGDA (Monomer-Polymer and Dajac Labs, 98% purity), di-*tert*-butyl peroxide (TBPO, Sigma-Aldrich, 97%), and silicon wafers (University wafers).

Depositions were conducted in a custom-built vacuum deposition system. The vacuum chamber’s pressure was monitored by a pressure transducer (MKS Baratron) and controlled using a downstream throttle valve (MKS Type T3BI). The deposition pressure was controlled from 100 to 1000 mTorr. The substrate temperature was controlled by a recirculating chiller/heater unit, a liquid-filled aluminum cold plate, and a thermal electric cooler (Type TEC1-12706). Power was supplied to the nichrome filament by a DC power supply (GW Instek, PSW 80-13.5). The volumetric flow rates of vapor-phase octamethylcyclotetrasiloxane (D4) and naphthalene were both controlled by mass flow controller (MKS Type 1150). The volumetric flow rates of vaporized GMA and EGDA were controlled using needle valves (Swagelok SS-4BMRG). The flow rate of TBPO was controlled by mass flow controller (HORIBA SEC-4400). The monomer and porogen’s degree of saturation was determined by the volumetric flow rates of each precursor, chamber pressure, and each component’s saturated vapor pressure at the corresponding substrate temperature. The saturated vapor pressure was calculated according to Antoine equation or Clausius–Clapeyron equation using the reported vapor pressure data collected from Scifinder^36^ and Chemspider.^26^

For a typical deposition, before the deposition commenced, the chiller was turned on and set to the desired substrate temperature to ensure a uniform temperature distribution over the entire substrate. The monomer and porogen precursors were preheated to generate sufficient vapor pressure; TBPO remained at room temperature. The gaseous flow of monomer and porogen was fed into the vacuum chamber for 10 min to stabilize the flow rates, and the chamber pressure was set at the operating pressure. After 10 min, the gaseous flow of TBPO was introduced into the chamber, and the filament was turned on. A deposition duration of 5 to 10 min was used. If thermal shock was required, the thermoelectric cooler was turned on, which lowered the substrate temperature to the target temperature within seconds. After deposition, all vapor flows were turned off, and nitrogen gas was flowed into the chamber to maintain the original operating pressure. The filament remained on for another 10 min to ensure that the film was cured. Finally, the chamber was opened, and the sample was collected without fully pumping down the deposition system. A 24 h annealing process at 60 °C and ambient pressure were implemented to remove any residual porogen or monomer in the sample. This annealing condition was determined to be sufficient for complete porogen removal, though a reduction of the annealing duration is possible through further optimization.

Deposited films were imaged using electron microscopy. SEM (Zeiss, AURIGA) was used to characterize the cross-sectional structure, surface morphology, and thickness of the porous thin film. A 4 nm thick platinum coating was sputtered onto deposited films prior to imaging to avoid electron accumulation. The porosity was calculated by employing an image analysis algorithm^31^ implemented in Python. Combining imaging methods like SEM with image analysis algorithms to calculate pore size and porosity is believed to be a straightforward and reliable characterization of surface topology.^41^ The image processing steps are described as follows: the pore and polymeric phases are differentiated through Otsu’s thresholding.^31^ Pixels with a brightness value below the brightness threshold are counted as part of the pore area. Porosity of the film is then calculated by dividing the total number of pixels of the SEM images by the number of pixels belonging to the pore area. The average pore size of the voided film is estimated through dividing the area of pores by the total number of pores. The number of pores was estimated by detecting the contours of each pores employing a gradient algorithm.^42^

The density was estimated by dividing the mass of the film by its volume. The mass was measured by an ultra-microbalance (Mettler Toledo, UMX2 Ultra-microbalance), and the volume was estimated using the product of surface area and film thickness.

FTIR spectra were collected on a Nicolet is50 FTIR spectrometer. The spectra of all polymeric coatings were collected in the transmission mode. The GMA, EGDA, naphthalene, and D4 precursors were characterized by attenuated total reflection (ATR) (Specac GoldenGate). The ATR correction algorithm in OMNIC 6.2 software was used to correct for the relative band intensity distortion and absolute band shifts in frequency. The resolution was set to 4 cm^–1^ for all experiments, and a total number of 64 scans were integrated to enhance the signal-to-noise ratio.

## Supporting Information Available

Flow chart depicting the refinement of deposition conditions, processed SEM images and pore size distribution histogram of SP-N1, -N2, -N3, and -N4 and of SP-D1, -D2, -D3, and -D4 before and after image processing, and FTIR spectra of octamethylcyclotetrasiloxane(D4), as-deposited SP-D4, and annealed SP-D4 (PDF)
