# Supplementary material for: Porous Polymer Films with Tunable Pore Size and Morphology by Vapor Deposition
Source: ACS Appl Polym Mater. 2022 Sep 20;4(10):7300–10. doi: 10.1021/acsapm.2c01032 (PMC9578110; doi:10.1021/acsapm.2c01032)
Supplement: Supplementary file 1 — ap2c01032_si_001.pdf [file ap2c01032_si_001.pdf]

Supporting Information

Porous polymer films with tunable pore size and morphology by vapor deposition

Ni Huo[a], Sheng Ye[b], Andrew J. Ouderkirk[b], Wyatt E. Tenhaeff[a]*

a. Department of Chemical Engineering, University of Rochester, Rochester, NY 14627, USA
b. Facebook Reality Labs, 9845 Willows Rd, Redmond, WA 98052, USA

Corresponding Author:
*Email: wyatt.tenhaeff@rochester.edu (W. E. Tenhaeff)

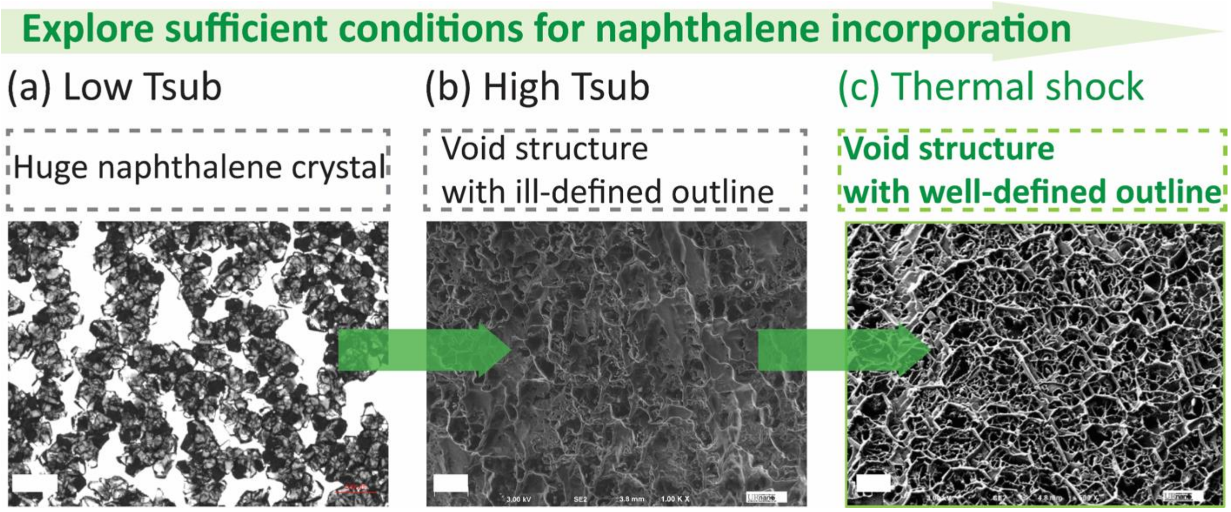

Figure S1. Flow chart depicting the refinement of deposition conditions. The effect of (a) low substrate temperature (T_{sub}) (b) high T_{sub} and (c) thermal shock process on film morphology is apparent from the SEM micrographs. Scale bars represent 200µm, 10µm, and 20µm respectively.

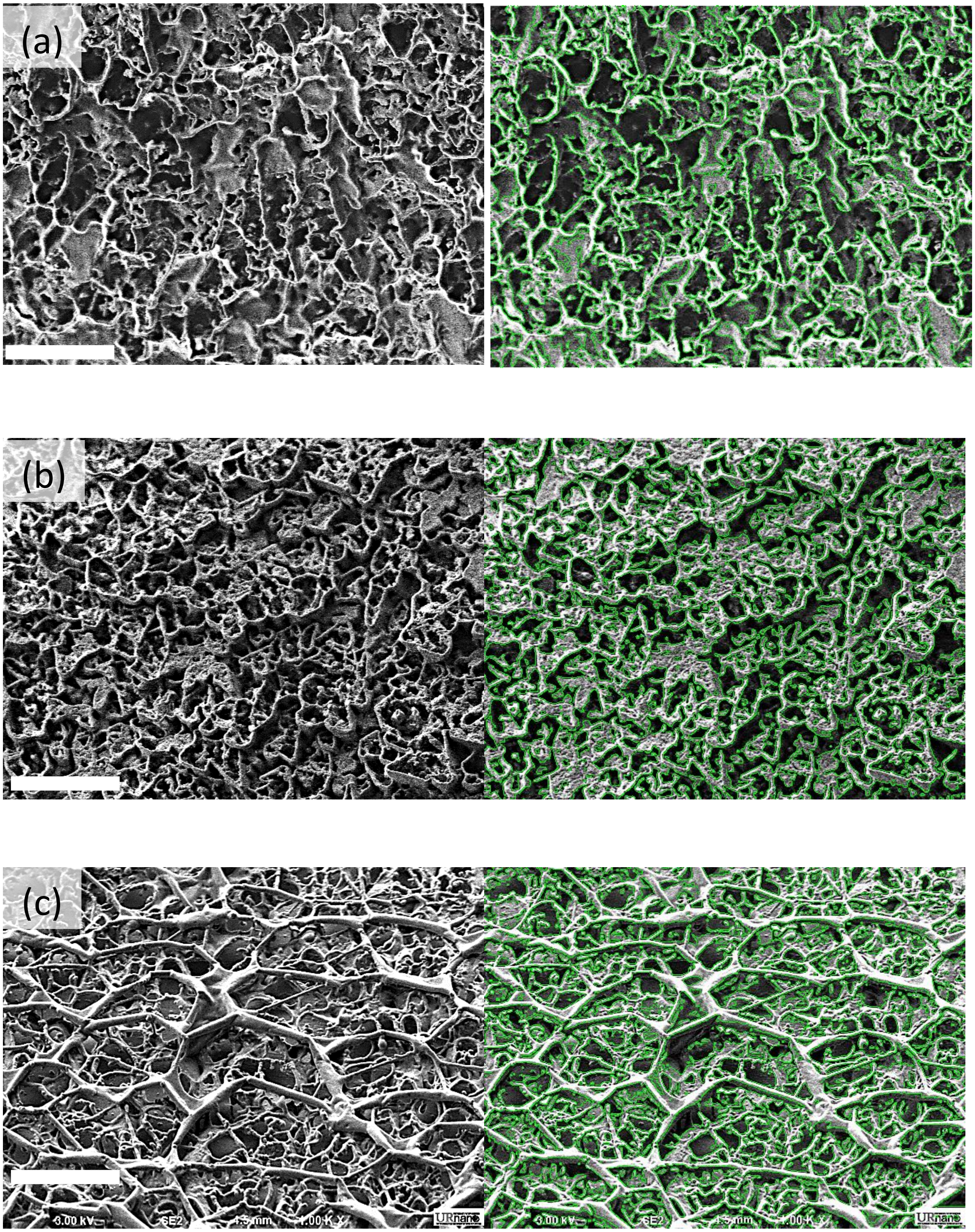
(a)
(b)
(c)
3.00 kV
SE2
4.5 mm
1.00 K X
URnano

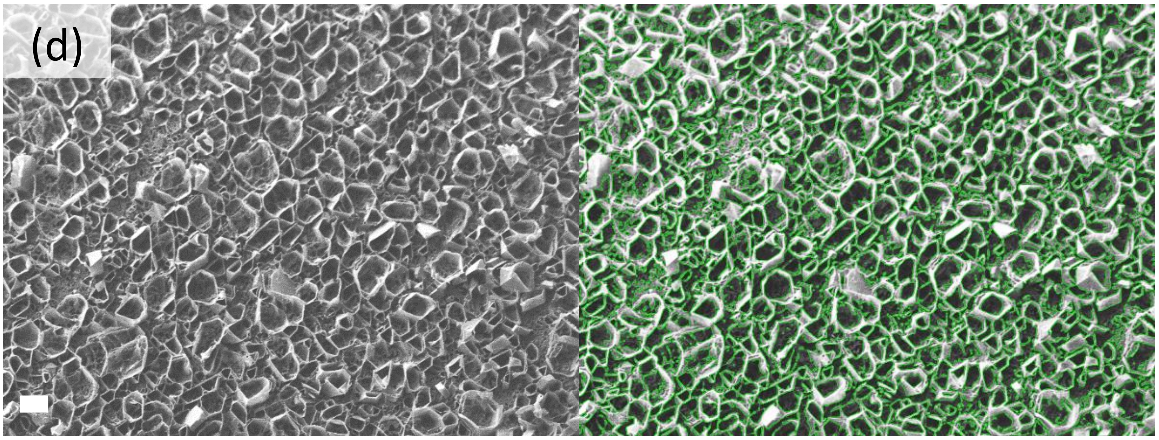

Figure S2. Processed SEM images of (a) SP-N1, (b) N2, (c) N3, and (d) N4 before and after image processing. The green lines indicate the contours of pores identified by edge detection algorithms.[1] All scale bars represent 20um.

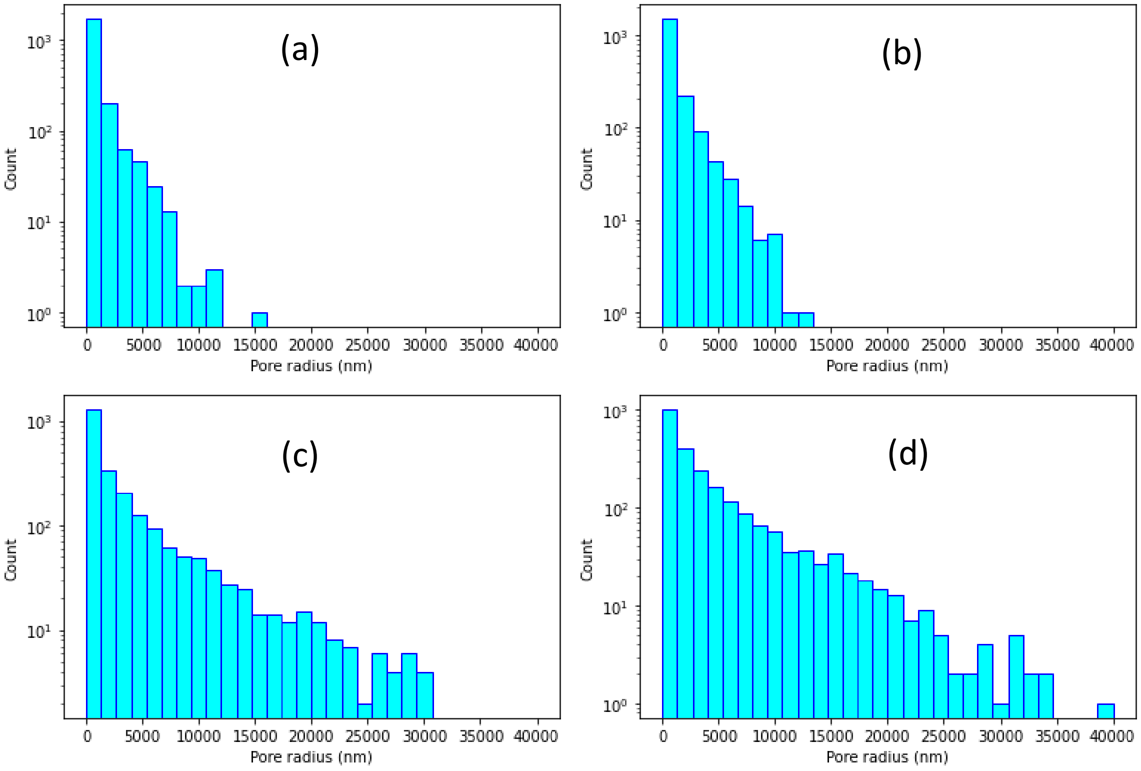

Figure S3. Pore size distribution histogram of (a) SP-N1, (b) -N2, (c) -N3, and (d) -N4.

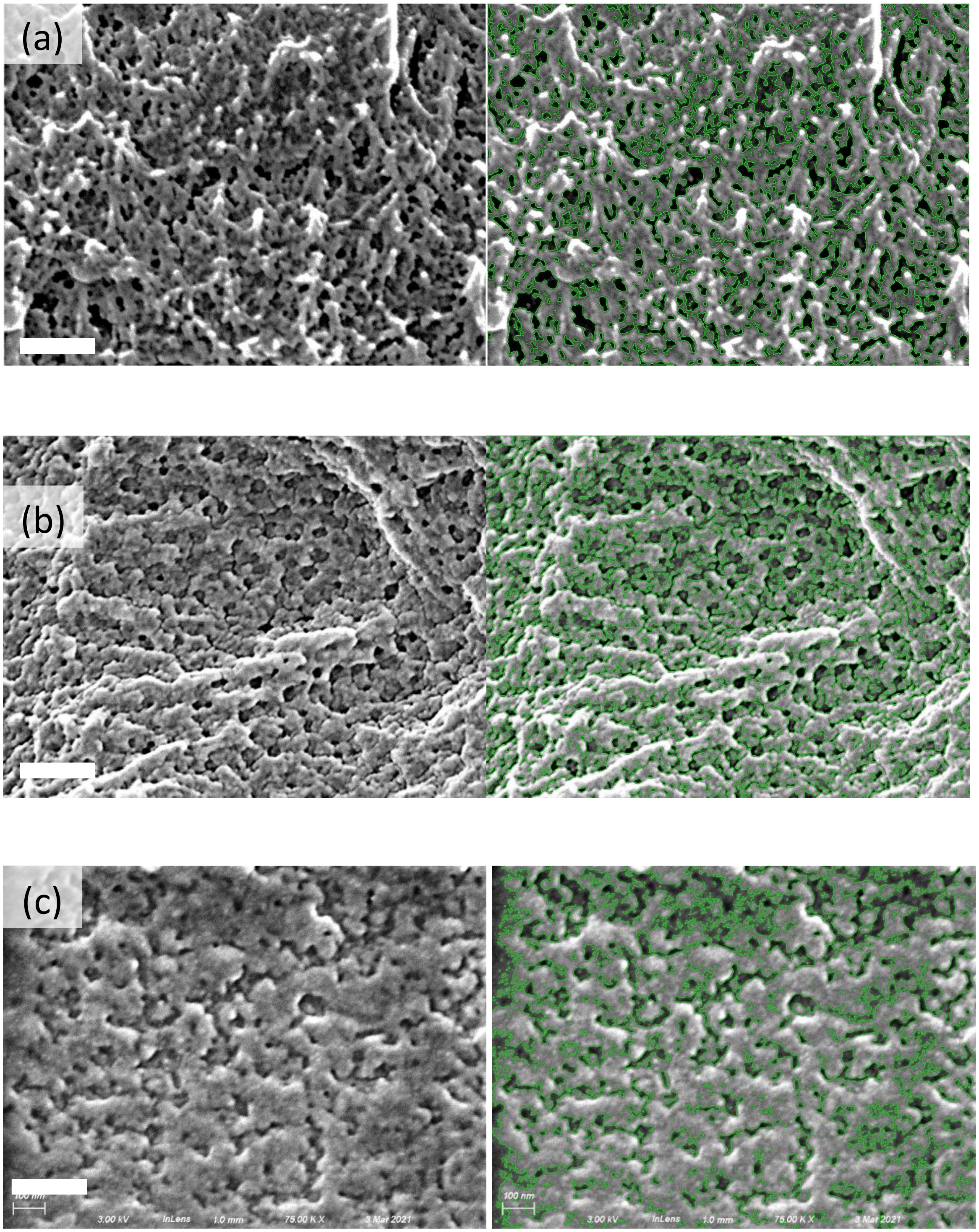
(a)
(b)
(c)
100 nm
3.00 kV
InLens
1.0 mm
75.00 K X
3 Mar 2021

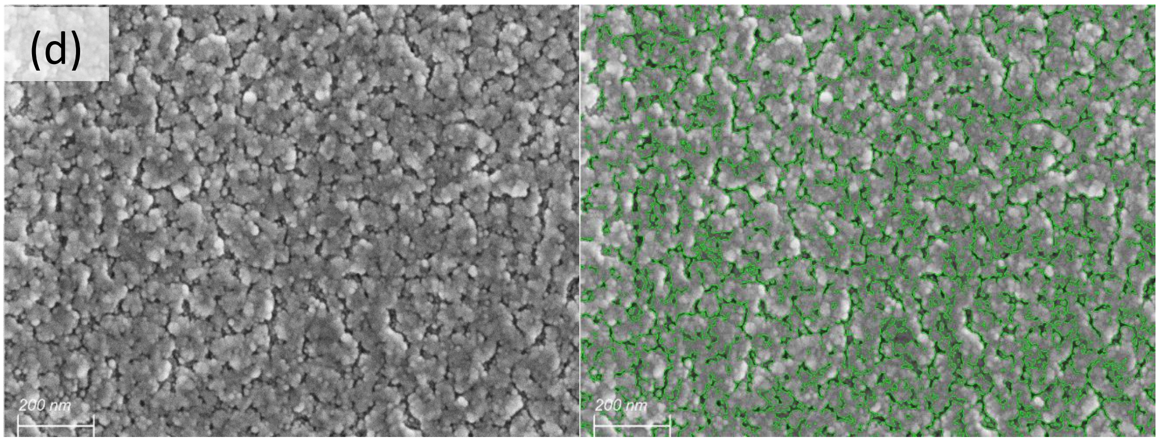

Figure S4. Processed SEM images of (a) SP-D1, (b) -D2, (c) -D3, and (d) -D4 before and after image processing. The green lines indicate the contours of pores identified by edge detection algorithms.[1] All scale bar represent 200 nm.

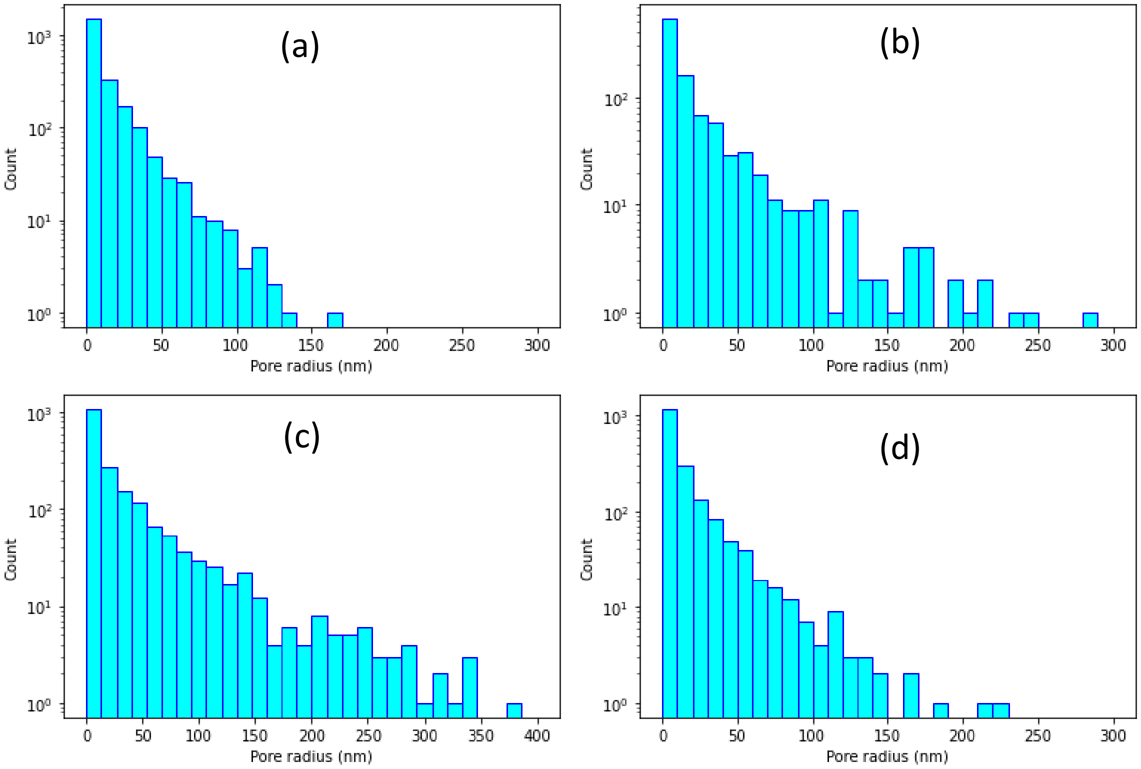

Figure S5. Pore size distribution histogram of (a) SP-D1, (b) -D2, (c) -D3, and (d) -D4.

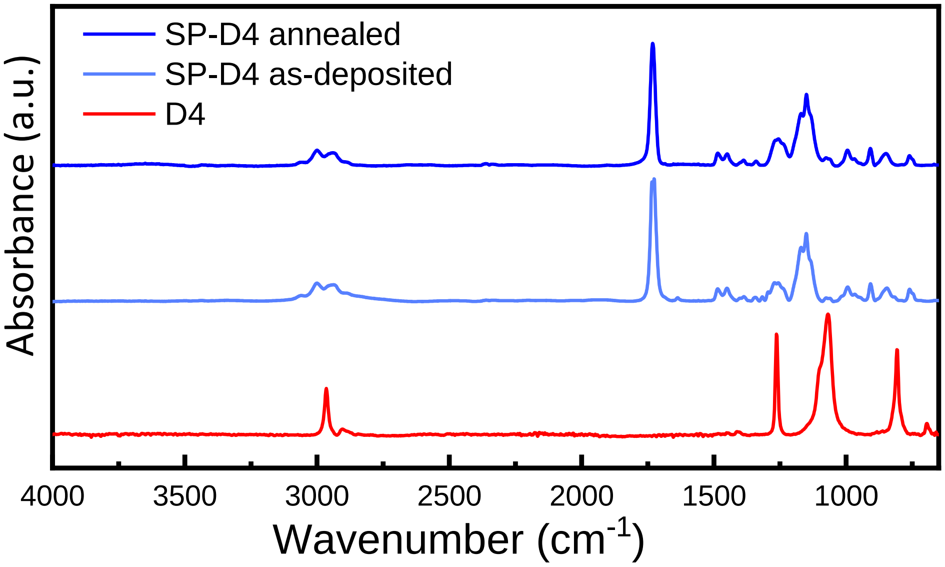

Figure S6. FTIR spectra of octamethylcyclotetrasiloxane(D4), as-deposited SP-D4 and annealed SP-D4.

References:

1. Image Processing - OpenCV. https://docs.opencv.org (accessed June 6, 2022).
